# Pediatricians’ Compliance to the Clinical Management Guidelines for Community-Acquired Pneumonia in Infants and Young Children in Pakistan

**DOI:** 10.3390/healthcare9060701

**Published:** 2021-06-09

**Authors:** Sadia Shakeel, Wajiha Iffat, Ambreen Qamar, Faiza Ghuman, Rabia Yamin, Nausheen Ahmad, Saqib Muhammad Ishaq, Márió Gajdács, Isha Patel, Shazia Jamshed

**Affiliations:** 1 Faculty of Pharmaceutical Sciences, Dow College of Pharmacy, Dow University of Health Sciences, Karachi 74200, Pakistan; sadia.shakeel@duhs.edu.pk (S.S.); wajiha.iffat@duhs.edu.pk (W.I.); 2 Department of Physiology, Dr. Ishrat Ul Ebad Khan Institute of Oral Health Sciences (DIKIOHS), Dow University of Health Sciences, Karachi 74200, Pakistan; ambreen.qamar@duhs.edu.pk; 3 Dow University Hospital, Dow University of Health Sciences, Karachi 74200, Pakistan; faizulmaram@hotmail.com; 4 Department of Pediatrics, National Institute of Child Health, Karachi 74200, Pakistan; rabia90001@gmail.com; 5 Jinnah Postgraduate Medical Centre, Department of Chest Medicine, Karachi 74200, Pakistan; drnausheenahmad@hotmail.com; 6 Scientific Assistant, Karachi Institute of Radiotherapy and Nuclear Medicine (KIRAN), Karachi 74200, Pakistan; saqibghazali93@gmail.com; 7 Faculty of Medicine, Institute of Medical Microbiology, Semmelweis University, 1089 Budapest, Hungary; gajdacs.mario@szte.hu; 8 Department of Pharmacodynamics and Biopharmacy, Faculty of Pharmacy, University of Szeged, 6720 Szeged, Hungary; 9 School of Pharmacy, Marshall University, Huntington, WV 25755, USA; pateli@marshall.edu; 10 Department of Clinical Pharmacy and Practice, Faculty of Pharmacy, Universiti Sultan Zainal Abidin, (UniSZA), Kuala Terengganu 21300, Malaysia; 11 Qualitative Research-Methodological Application in Health Sciences Research Group, Kulliyyah of Pharmacy, International Islamic University Malaysia, Kuantan 25200, Malaysia

**Keywords:** pediatricians, lower respiratory tract infections, community-acquired pneumonia, clinical management guidelines, Pakistan

## Abstract

Community-acquired pneumonia (CAP) is among the most commonly prevailing acute infections in children that may require hospitalization. Inconsistencies among suggested care and actual management practices are usually observed, which raises the need to assess local clinical practices. The current study was conducted to evaluate pediatricians’ compliance with the standard clinical practice guidelines and their antibiotic-prescribing behavior for the management of CAP in children. *Methods*: A descriptive cross-sectional study was conducted using a self-administered questionnaire; which was provided to pediatricians by the researchers. Statistical analysis was performed with SPSS 25 Statistics; χ^2^ tests (or Fisher-exact tests) with the *p*-value set at < 0.05 as the threshold for statistical significance. *Results*: The overall response rate was 59.2%. Male respondents were (*n* = 101; 42.6%), and the respondents (*n* = 163; 68.7%) were under 30 years of age. Amoxicillin (*n* = 122; 51.5%) was considered as the most commonly used first-line treatment for non-severe pneumonia, whereas a smaller proportion (*n* = 81; 34.2%) of respondents selected amoxicillin–clavulanate. Likewise, amoxicillin (*n* = 100; 42.2%) was the most popular choice for non-severe pneumonia in hospitalized children; however, if children had used antibiotics earlier to admission, respondents showed an inclination to prescribe a macrolide (*n* = 95; 40.0%) or second-generation cephalosporin (*n* = 90; 37.9%). More than 90% responded that children <6 months old with suspected bacterial CAP will probably receive better therapeutic care by hospitalization. Restricting exposure to the antibiotic as much as possible (*n* = 71; 29.9%), improving antibiotic prescribing (*n* = 59; 24.8%), and using the appropriate dose of antimicrobials (*n* = 29; 12.2%) were considered the major factors by the respondents to reduce antimicrobials resistance. *Conclusions*: The selection of antibiotics and diagnostic approach was as per the recommendations, but indication, duration of treatment, and hospitalization still can be further improved.

## 1. Introduction

Community-acquired pneumonia (CAP) is a commonly prevailing lower respiratory tract (LRT) infection that remains one of the major reason for early pediatric mortality, particularly in resource-limited countries [1,2]. A child having rapid, shallow breathing (tachypnea) and chest indrawing is considered to have “pneumonia”. CAP refers to pneumonia that is developed in a child in a community or outside of the healthcare system [2]. CAP is among the most well-known respiratory diseases requiring pediatric hospitalization. Inappropriate management of CAP patients or the delayed admission of patients to the intensive care unit (ICU) is revealed to be accompanied with augmented mortalities, and it is critical for general practitioners (GPs) to detect relevant patients suffering from severe pneumonia as quickly as possible [3]. The United States alone reports an incidence of 634 cases per 100,000 persons, leading to almost 1.5 million hospitalizations per annum [4]. However, in Pakistan, there are a lack of data on burden of pneumonia in terms of morbidity and mortality.

The World Health Organization (WHO) has defined the health profile of Pakistan by increase in maternal mortality, higher population growth rate, higher infant and child mortality, and increased disease burden. Optimal management of CAP is a challenging task particularly in resource-limiting countries such as Pakistan, which is lacking advanced healthcare facilities. Although the population in Pakistan had inadequate access to appropriate healthcare facilities, the number of medical graduates had been rising with a dramatic increase seen from 1300 in 1977 to 3800 in 1988 [5]. Presently, the doctor-to-patient ratio is 1:1300 in Pakistan, which is bigger than a baseline of 1:60,000 in 1947 [5]. Even with doctors graduating in such large figures, the shortage is still not addressed, and there is an irregular distribution of healthcare services among urban and rural regions [6]. In addition, the empiric use of antibiotics for acute respiratory infections is one of the leading factors of antibiotic misuse in developing countries. Inadequate evidence on potential pathogens and the local profile of bacterial drug resistance usually leads to poorly directed and needless antibiotic use [4]. Inadequate contemplation of the risks accompanying the unwanted use of antibiotics is a matter of serious concern. Boosting the rational use of antibiotics is a global priority, especially in Asian countries, where the non-prescription accessibility of antibiotics is commonly unobstructed [5]. The PIDS/IDSA recommendations for the management of CAP were generated to support clinicians in the management of CAP in children and to homogenize medicinal care [6]. These guidelines aim to decrease morbidity and mortality rates in children with CAP by improving the clinical care of these patients. However, there are some inconsistencies between actual management practices and recommendations, leading to the misuse of antibiotics, an increase in resistance rates of community-acquired pathogens, and extensive duration of hospitalizations [7].

Physicians contribute significantly to the management of CAP by immediate diagnosis, proper antibiotic regime, and by recognizing risk factors. Discrepancies among suggested care and actual management strategies by the physicians are usually observed, which raises the need to assess our local clinical practices. In Pakistan, inadequate exploration has been conducted on CAP, and the current study was the first one conducted with the aim to evaluate pediatricians’ compliance to the standard clinical practice guidelines and their antibiotic-prescribing behavior for the management of CAP in children.

## 2. Materials and Methods

### 2.1. Study Design

A descriptive cross-sectional study was conducted during the period of two months between December 2020 and January 2021 using a self-administered questionnaire, which was provided to pediatricians by the researchers.

### 2.2. Study Population and Sample Size Calculation

The pediatricians and physicians—particularly those working in pediatric wards—in 4 public and 2 private tertiary care hospitals of Karachi were considered as the target population of the study. Karachi is the biggest city in Pakistan and the twelfth largest city in the world. It is the capital of the Sindh province of Pakistan. Respondents were selected by the convenience sampling technique. The size of the study sample was calculated with the Raosoft sample size calculator (Raosoft Inc.^®^, Seattle, WA, USA) [8] by using the following formula:

x = Z(^c^/_100_)^2^ r(100 − r)

n = ^N x^/_((N−1)E^2^ + x)_

E = Sqrt[^(N − n)x^/_n(N − 1)_]

where r is the fraction of responses, N is the population size, n is the sample size, E is the confidence interval, c is the confidence level, and Z(c/100) is the critical value for the confidence level.

### 2.3. Inclusion and Exclusion Criteria

The following respondents were included in the study:

- pediatricians or physicians working in pediatric settings of the hospital, including neonatal intensive care unit (NICU)/neonatal care, pediatric intensive care (PIC), pulmonology, emergency medicine, infectious diseases, outpatient clinics, general pediatrics, and the private pediatric clinics and
- if they agreed to participate in the study. When potential respondents were approached, they were briefed about the objectives of the study and the time required to complete the survey. They were informed that no incentives will be presented to them for their contribution and that participation is voluntary.

### 2.4. Study Instrument

A 42-item questionnaire was developed, based on a comprehensive review of the literature [9], the clinical practice recommendations by the Pediatric Infectious Diseases Society (PIDS) [10], and the Infectious Diseases Society of America (IDSA) [6] under the guidance of the opinions of an expert panel, including three senior physicians and two infectious disease specialists. On concluding the content validity, the questionnaire was pre-tested in a small sample of physicians (*n* = 30), to evaluate the clarity and transparency of the individual questionnaire items (face validity). The Hoyt method λ 3 {\displaystyle \lambda _{3}} reliability scale was applied, and the value of reliability was found to be 0.783. Then, the questionnaires were circulated via email or direct correspondence among the respondents, after apprising their motivation to participate in this research. The questionnaire has 4 questions related to demographic characteristics, and 21 questions addressed their agreement with the recommended clinical practice guidelines. The questionnaire includes 17 items to analyze the respondents’ adherence toward the guidelines in their practice using a 5-point Likert-scale ranging from 1 = strongly disagree to 5 = strongly agree. Additionally, the questions were included regarding the respondents’ preferences and their practices for antibiotic treatment of children with CAP, indications and duration of CAP hospitalization, IV antibiotic use, and ICU admission.

### 2.5. Ethical Considerations

The study was conducted agreeing with the recommendations of the Declaration of Helsinki and approved by the Institutional Review Board of Liaquat College of Medicine and Dentistry, Darul Sehat Hospital, Karachi, Pakistan (Reference No. DSH/IRB/2021/0028). The written consent was taken from the respondents before the study.

### 2.6. Data Analysis

The data were evaluated by Statistical Package for the Social Sciences^®^ (SPSS) for Windows version 25.0 (IBM Corporation, Armonk, NY, USA). All categorical variables were described using frequencies (n) and proportions (%). Data were statistically analyzed with χ^2^ tests (or Fisher-exact tests) with the p-value set at < 0.05 as the threshold for statistical significance.

## 3. Results

### 3.1. Demographic Characteristics

In this study, 400 survey forms were distributed among the pediatricians and physicians working in pediatric wards belonging to different tertiary care hospitals in Karachi. Two hundred and thirty-seven (*n* = 237) pediatricians and physicians consented to participate in the research. The response rate observed was 59.2%. Out of the respondents, male respondents were in the minority (*n* = 101; 42.6%), while (*n* = 163; 68.7%) of respondents were under 30 years of age (Table 1). The majority of the respondents (*n* = 151; 63.7%) were serving in public hospitals, while a little over half (*n* = 123; 51.8%) of respondents had 1–5 years of clinical experience. The respondents (*n* = 225; 94.9%) routinely prescribe antibiotics to the patients. However, the response was significantly associated with the age (*p* = 0.014), type of working hospital (*p* = 0.02), and experience (*p* = 0.001) of respondents. More than two-thirds (*n* = 160; 67.5%) of respondents stated that they would not treat children with antibiotics if a viral LRT infection is presumed. The majority of the respondents (*n* = 180; 75.9%) preferred the oral route of antibiotic administration in children compared to intravenous (IV) administration.

### 3.2. Respondents’ Preferences for Antibiotic Treatment in a CAP Child

Table 2 summarizes the respondents’ preferences for antibiotic treatment in a CAP child. Amoxicillin (*n* = 122; 51.5%) was considered as the most preferred first-line treatment for non-severe pneumonia, whereas other respondents (*n* = 81; 34.2%) opted for amoxicillin–clavulanate. Likewise, amoxicillin (*n* = 100; 42.2%) was the most popular choice for non-severe pneumonia in hospitalized children; however, if children had previously received antibiotics prior to admission, respondents showed an inclination to instead prescribe a macrolide agent (*n* = 95; 40.0%) or a second-generation cephalosporin (*n* = 90; 37.9%). The response varies with the type of working hospital of respondents; those who were working in a private setting were more likely to use macrolides (*p = 0.011*). In a child hospitalized with severe CAP, a third-generation cephalosporin (ceftriaxone or cefotaxime) was generally chosen by respondents (*n* = 155; 65.4%) if a child had not used antibiotics earlier to admission, together with gentamicin as an add-on medication. The response rate varies with the experience of respondents (*p = 0.021*). Likewise, a third-generation cephalosporin (ceftriaxone or cefotaxime) was selected by the majority of the respondents (*n* = 184; 77.6%) if a child had received antibiotics prior to hospital admission.

### 3.3. Respondents’ Practice for the Duration of CAP Hospitalization and IV Antibiotic Use

Table 3 summarizes respondents’ self-reported existing practices, depicting the average length of CAP hospitalization and the administration of IV antibiotics. The respondents specified that they would discharge a child with non-severe CAP within 5 days (*n* = 143; 60.5%) but keep a child with severe CAP hospitalized for 6–7 days (*n* = 115; 48.5%). Older respondents were more likely to keep a child hospitalized if they are affected by severe pneumonia for 8–10 days (*p = 0.016*). In case of a child with severe pneumonia, more than half of respondents (*n* = 128; 54.0%) provided IV antibiotics for 6–7 days, while only three respondents indicated an inclination for switching to oral antibiotics. More than 90% stated that blood cultures should normally not be taken in a completely vaccinated child with CAP, receiving treatment in an outpatient setting. Around 92% of respondents said that specific and sensitive tests for the prompt identification of influenza and other respiratory tract viruses must be conducted for the assessment of a CAP child.

The national guidelines (*n* = 180; 75.9%) followed by a local experts’ opinion (*n* = 29; 12.2%) were the more persuasive factors for respondents’ antibiotic prescribing behavior. (see Table 4). Clinical assessment (*n* = 197; 83.1%) followed by a complete course of IV antibiotics (*n* = 19; 8.0%) were the major factors influencing the respondents’ decision to discharge a child from the hospital. Around 90% of the respondents stated that a hospitalized CAP child could be discharged safely when they show complete improvement in CAP symptoms, including reduced fever for at least 12–24 h, the normal activity level, a healthy appetite, stable mental status, and consistent pulse oximetry measurements >90% in ambient air for at least 12–24 h.

### 3.4. Respondents’ Perceived Indications for CAP Hospitalization, Antibiotic Therapy, and ICU Admission

Table 5 showed the respondents’ perceived indications for CAP hospitalization and the administration of antibiotic therapy for CAP. Almost all the respondents stated that a moderate-to-severe CAP child must be hospitalized to achieve adequate care; however, more experienced respondents were more likely to believe that hospitalization is necessary to provide sufficient therapeutic care to the patient (*p = 0.026*). More than 90% opined that children of age <6 months with suspected bacterial CAP may get better therapeutic care by hospitalization. The majority (*n* = 210; 88.6%) of the physicians responded that amoxicillin must be used as the first choice for fully immunized infants and toddlers who were previously healthy, with minor-to-moderate bacterial CAP. The respondents (*n* = 190; 80.1%) stated that vancomycin or clindamycin (depending on susceptibility data) must be given along with the β-lactam therapy, if clinical presentation indicates a *Staphylococcus aureus* infection. More than 75% of respondents agreed that amoxicillin appropriately provides coverage for *Streptococcus pneumoniae,* and influenza antiviral treatment should be directed as soon as possible to a child with moderate-to-severe CAP consistent with an influenza virus infection. More than 90% (*n* = 215) of respondents indicated the need for ICU admission if a child needs invasive ventilation, having altered mental status and depicting other altered clinical, radiological, and laboratory findings. The respondents with the higher age (*p = 0.037*), greater experience (*p = 0.021*), and working in private hospital (*p = 0.002*) showed better knowledge regarding the child’s need for ICU admission.

### 3.5. Respondents’ Perceived ways of Reducing Antimicrobials Resistance through Appropriate Management

**Figure 1:**
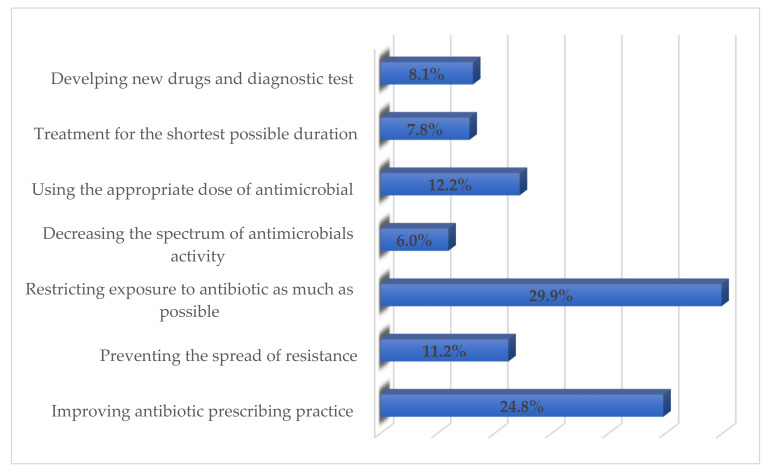
Respondents’ perceived ways of reducing antimicrobials resistance.

**Figure 2:**
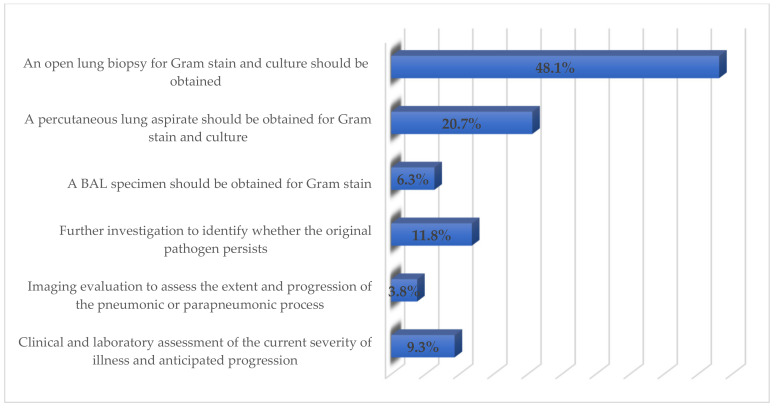
Respondents’ perceived management of the child not responding to treatment.

Restricting exposure to the antibiotic as much as possible (*n* = 71; 29.9%), improving antibiotic prescribing (*n* = 59; 24.8%), and using the appropriate dose of antimicrobials (*n* = 29; 12.2%) were considered the major factors by the respondents to reduce antimicrobials resistance (Figure 1). The majority of respondents (48.1%) opined that an open lung biopsy should be conducted for Gram-stain and culture in a child not showing any improvement within 48–72 h of treatment (Figure 2). More than 90% stated that all children and adolescents should be vaccinated once a year for the influenza virus to prevent the development of pediatric CAP.

## 4. Discussion

The current study was executed to investigate the physicians’ compliance to the standard clinical practice recommendations for managing pediatric CAP. The response rate of the current study was 59.2%, which may be considered appropriate in light of the literature findings. A similar lower response rate of physicians was observed in another study [11]. Studies have shown that the response rates are usually lower for physicians as compared to general community surveys, which may be due to physicians’ tough work schedules and an increase in the frequency of being approached for studies [12,13].

In the present study, the majority of the respondents commonly prescribe antibiotics to the patients. Therefore, it is significant to evaluate the physicians’ practice of using antibiotics, along with factors influencing their prescribing behavior to develop future approaches to increase the rational use of antibiotics [14]. However, the majority of respondents stated not to give an antibiotic to a child with a presumed viral LRT infection; the principal reason for this was that they knew the adverse effects accompanying the use of antibiotics in children. The misuse of antibiotics is related to preventable adverse drug effects and greater healthcare expenses and leads to the increasing global crisis of antimicrobial resistance [14]. In addition, while some novel agents have been authorized recently for the use of treating CAP (delafloxacin, lefamulin), nevertheless, none of these agents may be used in the pediatric population [14]. The healthcare professionals must contemplate their responsibility in educating the community to inhibit the irrational usage of antibiotics. In this study, the majority of the respondents preferred the oral route of antibiotics in children, which is parallel to the findings of another study in which the physicians favored using oral over intravenous or intramuscular antibiotic injections [9].

The PIDS/IDSA guiding principles publication in 2011 were set to permit improved clinical effects and decrease management discrepancies [6]; several studies inspected pediatricians’ adherence to them. In our study, an inclination for broad-spectrum antibiotics was observed even though using antibiotics such as amoxicillin–clavulanate or later-generation cephalosporin administered empirically do not propose any benefit over amoxicillin [15]. Among the surveyed respondents, third-generation cephalosporin was preferred for a child with severe CAP regardless of preceding exposure to the first-line therapy with antibiotic. Another study reported that treatment with the narrow-spectrum antibiotics was not less significant to broad-spectrum antibiotics in all measurable clinical outcomes together with the length of stay and duration of fever [16]. Many pediatricians stated that a cephalosporin must be used if a child does not show an improvement to first-line therapy with oral amoxicillin or IV ampicillin for non-severe and severe pneumonia, respectively. Younger respondents usually follow the local practices, even though they do not conform with standard pediatric CAP management recommendations [17]. It is observed that physicians considered broader-spectrum or novel antibiotics as more effective, which is an imprint that is frequently reinvigorated by promotion from pharmaceutical organizations [18]. The national guidelines followed by local expert opinion were the respondents’ reported persuasive factors for antibiotic prescribing behavior in the present study. At times, the parent beliefs, along with their expectations and demands, contribute significantly to inappropriate antibiotic-prescribing by physicians. It is observed that due to the self-perceived seriousness of the infection, sometimes, the parents who get more anxious about their child’s disease are likewise more inclined to use antibiotics. Patients who expect to receive antibiotics were prescribed antibiotics more frequently comparing to those who were not expecting them. For that reason, the inappropriate prescribing and promotion of antibiotics in the community might be due to both the patients’ demands and the profit interest of the healthcare providers, which may jeopardize the healthcare needs and safety of the patients. The parallel findings were reported by another study, in which some physicians stated that pressure from patient’s family members persuaded them to prescribe antibiotics [9]. This finding was reinforced by a Hungarian study, where 25.0% of the respondents admitted that the temperament of patients (or parents) significantly influences their antibiotic-prescribing practices; additionally, 69.2% think that much more effort should be put into providing the point-of-care tests (POCT) to help physicians to better assess the presence of an indication for antibiotic therapy [19]. In the current study, around 92% stated that specific and sensitive examinations for the prompt identification of influenza and other respiratory tract viruses must be conducted in the assessment of a child having CAP. It is evident that enhanced timely information of likely pathogens at the initial stage of therapeutic care and the probable susceptibility of bacterial pathogens might support in guiding antibiotic-prescribing choices and consequently prevent avoidable antibiotic use [19]. More than 90% of respondents in the current study stated that blood cultures should normally not be conducted in a completely vaccinated child with CAP getting treatment in the outpatient setting. However, the blood cultures should be taken in a child who needed hospitalization for presumed moderate-to-severe bacterial CAP, specifically those with the complex clinical presentations. It is neither easy nor cost-effective to detect microbial etiology in all pediatric patients presented with LRT infections in primary care due to sampling challenges, inadequate diagnostic facilities, and the limited clinical effectiveness of getting a result once empirical treatment choice has been made [20].

The current study revealed the inclination of respondents to provide IV antibiotics for 6–7 days in a child with severe pneumonia and less preference for switching to oral antibiotics. As per the American CAP treatment recommendations, there is no suggested rigid length of treatment; however, a 10-day antibiotic course is considered as adequate and could be protracted when there is a probability of certain pathogens or when radiological or clinical conditions get worse during a hospital stay [6]. The WHO recommends a shorter antibiotic course for children aged <5 years [21]. Numerous studies on the management of pediatric pneumonia revealed no major difference among short and long-duration courses neither in the measurement of clinical outcomes nor in management failure or recurring rates [22,23,24,25]. In the current study, more than 90% opined that a child of age <6 months with presumed bacterial CAP could get better therapeutic care during hospitalization. The clinical assessment followed by a complete IV antibiotic course were the major factors that influenced respondents’ decision to discharge a child from the hospital in the current study. Although physicians specified that they would generally discharge a child with non-severe pneumonia within 5 days, management with oral antibiotics (if necessary) at home has been revealed to be similarly effective [23]. The commonly practiced duration of hospitalization by the respondents for severe pneumonia was 6–7 days, which corresponds with a retrospective study where the average stay at the hospital was 7–6 days for acute respiratory infection [17]. Regardless of a common predilection for oral antibiotics, the respondents showed a willingness to complete a complete course of IV antibiotics in children with severe pneumonia. The significance of a timely oral switching strategy, which decreases hospital stay, secondary health problems, and other healthcare expenses, has been confirmed in several studies [24,25]. Yet, extended hospitalization for the IV antibiotics administration remains a general practice in the Asian region countries. By reducing needless hospitalization for IV antibiotics, major health expenses may be saved [26]; however, none of the study respondents confessed to generating revenue from prescribing antibiotics. In some countries, drug prescriptions normally increase a doctor’s earnings, and the exclusion of these profitable inducements accompanied by improved regulation can significantly decrease the misuse [27]. China has declared a strategy to preclude hospitals and physicians from earning from unnecessary use of antibiotics [28]. Almost all respondents comprehended that improved guidelines are necessary to fix the needless use of antibiotics in Pakistan; however, antibiotics remain continually available over the counter, and no limitations on their accessibility have been proclaimed to date. The problem of precisely differentiating viral and bacterial pneumonia is a key driver of the appropriate use of antibiotics. Respondents opined that the availability of enhanced microbiological facilities would decrease antibiotics use, and parallel findings were reported by another study [29]; they have indicated that despite a known respiratory virus, some respondents would still prescribe antibiotics due to bacterial co-infection concerns. In the current study, limiting exposure to an antibiotic was considered to be the most beneficial approach of reducing antimicrobial resistance. Other studies reported antibiotic stewardship programs, access to better microbiology services, and rapid influenza tests as the significant factors to decrease antibiotic prescriptions [30,31]. Another study revealed that a C-reactive protein test can progressively reduce avoidable antibiotic use [32]. However, it is certain that due to the lack of local surveillance data to provide evidence on drug resistance patterns of commonly prevailing respiratory pathogens, physicians are more likely to overuse empirical broad-spectrum antibiotics [33].

This is the first study conducted to formally assess pediatrician compliance toward the clinical practice recommendations for managing a child affected by CAP in Pakistan. However, the current study is limited, as it has only measured respondents’ self-perceived existing practices and has been directed to physicians working in the city of Karachi. The majority of respondents were less than 30 years of age and so were less experienced. The study was conducted on a limited number of pediatricians of Karachi, and the outcomes could not be generalized for the entire pediatricians of Pakistan or in other parts of the world. Due to the hectic clinical schedule, many respondents may have rushed to complete their surveys and spent a nominal amount of time in profound consideration. However, this prompt comprehensive response might have greater accurateness as the impulsiveness of replies must have condensed social desirability biases. Despite these limitations, it may be assumed that the responses may be comparable to the observed physicians’ practices in other studies.

## 5. Conclusions

The outcomes revealed that the selection of antibiotics and diagnostic approach was as per the recommendations, but indication, duration of treatment, and hospitalization still could be further improved. The respondents knew that there is a need to decrease preventable antibiotic use and to encourage timely oral switch as applicable. They emphasize the need for antibiotic stewardship, better drug regulation, and the availability of advanced microbiological services throughout Pakistan. The results have potentially prepared a platform to identify the discrepancies between actual management practices and standard recommendations that might be useful for local health authorities in developing future strategies to homogenize and provide optimum patient care.

## Figures and Tables

**Table 1:** Demographic characteristics of respondents.

Characteristics	Frequency (*n*; %)	
Gender	Male	101 (42.6)
	Female	136 (57.3)
Age	<30 years	163 (68.7)
	30–39 years	69 (29.1)
	40–49 years	5 (2.1)
Type of Working Hospital	Private	86 (36.2)
	Public sector	151 (63.7)
Experience	<1 year	62 (26.1)
	1–5 years	123 (51.8)
	5–10 years	47 (19.8)
	10–15 years	4 (1.6)
	>15 years	1 (0.4)
Use antibiotics extensively in clinical practice	Yes	225 (94.9)
	No	12 (5.0)
Preferable route of antibiotic administration	Oral	180 (75.9)
	Intravenous (IV)	45 (18.9)
	Intramuscular (IM)	12 (5.0)
Would you manage a child with a presumed viral LRT infection with antibiotics?	Yes	77 (32.4)
	No	160 (67.5)
Do you know the adverse effects associated with the use of antibiotics in children?	Yes	231 (97.4)
	No	5 (2.1)

**Table 2:** Respondents’ preferences for antibiotic treatment in a CAP child.

Scenario	Antibiotic Choice			
	First Priority, *n* (%)	Second Priority, *n* (%)	Third Priority, *n* (%)	Other Priorities, *n* (%)
	Non-Severe Pneumonia			
Outpatient	Amoxicillin	Amoxicillin clavulanate	Macrolide	
	122 (51.5)	81 (34.2)	25 (10.5)	9 (3.7)
Inpatient		**Non-Severe Pneumonia**		
Without previous use of antibiotic	Amoxicillin	Amoxicillin clavulanate	Macrolide	
	100 (42.2)	93 (39.2)	33 (13.9)	11 (4.6)
With previous use of antibiotic	Macrolide	Cephalosporin 2nd generation	Amoxicillin clavulanate	
	95 (40.0)	90 (37.9)	44(18.5)	8 (3.3)
		**Severe Pneumonia**		
Without previous use of antibiotic	Cephalosporin (3rd generation)	Ampicillin or penicillin G	Gentamycin	
	155 (65.4)	62 (26.1)	11 (4.6)	9 (3.7)
With previous use of antibiotic	Cephalosporin (3rd generation)	Ampicillin or penicillin G	Gentamycin	
	184 (77.6)	26 (11.0)	15 (6.3)	12 (5.0)

**Table 3:** Respondents’ practice for the duration of CAP hospitalization and intravenous antibiotic use.

Length of Hospitalization and Intravenous Use of Antibiotic	No. of Days	Frequency (*n*; %)
Duration of hospital stay in non-severe pneumonia	<3 days	66 (27.8)
	3–5 days	143 (60.5)
	6–7 days	21 (8.8)
	8–10 days	7 (2.9)
Duration of hospital stay in severe pneumonia	<5 days	14 (5.9)
	6–7 days	115 (48.5)
	8–10 days	73 (30.8)
	11–14 days	35 (14.6)
IV antibiotics to a child with severe pneumonia	<3 days	1 (0.4)
	3–5 days	82 (34.5)
	6–7 days	128 (54.0)
	8–10 days	26 (10.9)

**Table 4:** Elements that influenced respondents’ antibiotic prescribing behavior and decision to discharge a CAP child.

Decision	Strength of Influencing Factor			
	First	Second	Third	
Antibiotic prescribing behavior	National guidelines	Local Expert opinion	Best available drug	Other factors
	180 (75.9)	29 (12.2)	21 (8.9)	7 (2.9)
Hospital discharge	Clinical Assessment	Complete IV Antibiotic course	Parental request	Other factors
	197 (83.1)	19 (8.0)	9 (3.8)	12 (5.0)

**Table 5:** Respondents’ perceived indications for CAP hospitalization and antibiotic therapy.

Statement	Strongly Agree/Agree *n* (%)	Neutral *n* (%)	Disagree/Strongly Disagree *n* (%)
**Child or Infant with CAP who Must Be Hospitalized**			
A child with moderate to severe CAP must be hospitalized for better clinical care	235 (99.1)	0 (0)	2 (0.8)
Children of age <6 months with suspected bacterial CAP are likely to get better therapeutic care by hospitalization	214 (90.2)	20 (8.4)	3 (1.2)
Children with suspected CAP caused by an increased virulence pathogen, for instance, community-associated methicillin-resistant *Staphylococcus aureus* (CA-MRSA)	204 (86.0)	19 (8.0)	9 (3.7)
Children who lack vigilant observation at home or incapable of following the recommended therapy	192 (81.0)	27 (11.3)	13 (5.4)
**Anti-Infective Treatment**			
Antibiotic is not compulsory for a preschool-aged CAP child, as commonly, the infection is caused by virus.	172 (72.6)	31 (13.1)	34 (14.3)
Amoxicillin must be used as the first choice for immunized infants and toddlers who were previously healthy and have minor to moderate bacterial CAP.	210 (88.6)	17 (7.1)	9 (3.7)
Amoxicillin provides suitable coverage for *Streptococcus pneumoniae*.	185 (78.0)	29 (12.2)	23 (9.7)
Macrolide antibiotics must be given for the management of a child with CAP caused by atypical pathogens in an outpatient setting.	185 (78.0)	38 (16.0)	13 (5.4)
Influenza antiviral treatment should be directed as prompt as possible to a child with moderate to severe CAP due to the influenza virus.	179 (75.5)	44 (18.5)	14 (5.9)
Ampicillin or penicillin G should be given to the appropriately immunized child admitted in hospital with CAP due to *S. pneumoniae.*	168 (70.8)	40 (16.8)	29 (12.2)
Empiric treatment with a third-generation parenteral cephalosporin should be given for hospitalized infant or child who is not completely vaccinated, or for an infant or child with a life-threatening condition	186 (78.4)	43 (18.1)	8 (3.3)
Macrolide along with a β-lactam antibiotic must be given to the hospitalized child for whom *Chlamydophila pneumoniae* and *Mycoplsma pneumoniae* are major concerns.	166 (70.1)	51 (21.5)	15 (6.3)
Vancomycin or clindamycin should be provided along with β-lactam treatment if the infection is due to *Staphylococcus aureus*.	190 (80.1)	36 (15.1)	11 (4.6)
A child on appropriate therapy must reveal indications of improvement within 48–72 h.	182 (76.7)	33 (13.9)	22 (9.2)
A child whose illness worsens after admission or who did not show any progress within 48–72 h needs additional examination.	206 (86.9)	28 (11.8)	3 (1.2)
Outpatient parenteral antibiotic therapy should be given only to those children who do need nursing attention in an acute care setting; however, their ongoing parenteral treatment is necessary.	178 (75.1)	37 (15.6)	17 (7.1)
Switching to oral therapy whenever possible is preferential to parenteral outpatient treatment.	183 (77.2)	41 (17.2)	13 (5.4)

## Data Availability

Not applicable.
